# Type IIIb endoleak due to stent suture line fabric breakage in the Endurant stent graft: a case report

**DOI:** 10.1186/s40792-022-01415-8

**Published:** 2022-04-19

**Authors:** Satoshi Takahashi, Toshiya Nishibe, Masaki Kano, Shinobu Akiyama, Toru Iwahashi, Hitoshi Ogino

**Affiliations:** Department of Cardiovascular Surgery, Tokyo Medical University, 6-7-1 Nishi-shinjuku, Shinjuku-ku, Tokyo 160-0023 Japan

**Keywords:** Stent graft, Endoleak, Fabric breakage

## Abstract

**Background:**

Early type IIIb endoleak is a very rare complication of endovascular aneurysm repair (EVAR).

**Case presentation:**

An 87-year-old man was diagnosed with infrarenal abdominal aortic aneurysm. The patient underwent EVAR using the Endurant stent graft. Postoperative color duplex ultrasound revealed a regular row of pulsatile blood flow from the main body and left leg. The blood flow appeared to be bleeding from the stent suture lines because of its regularity. Type IIIb endoleak was suspected due to stent suture line fabric breakage but was not treated surgically or endovascularly because of the patient’s poor general health status. Six months later, contrast-enhanced CT demonstrated a deformation and enlargement of the aneurysm sac as well as an oozing of the contrast medium on the main body and left limb. Thereafter, he died of a subdural hematoma due to a fall. Autopsy showed no visible abnormal erosion or holes on the graft fabric, suggesting that suture line fabric breakage may have existed during the manufacturing process.

**Conclusions:**

Although rare, type IIIb endoleaks can occur even in the perioperative period after EVAR. Early type IIIb endoleaks may not resolve spontaneously and should be treated promptly, if possible.

**Supplementary Information:**

The online version contains supplementary material available at 10.1186/s40792-022-01415-8.

## Introduction

Type IIIb endoleak, defined as a fracture or tear in the graft material [1], is a rare complication of endovascular aneurysm repair (EVAR). Type IIIb endoleak is a high-pressure, high-risk leak [2] that leads to rapid growth of the aneurysmal sac and rupture [3]. Type IIIb endoleaks have been reported to occur as early as during operation to beyond 16 years postoperative, emphasizing the need for continuous surveillance [4, 5].

Herein, we report a rare case of type IIIb endoleak, likely due to stent suture line breakage and detected by color duplex ultrasound in the perioperative period, and we discuss the mechanism of occurrence and the fate of fabric breakage in early type IIIb endoleak.

## Case report

An 87-year-old man presented with appetite loss and was referred to a neighborhood hospital. His medical history included angina pectoris after percutaneous coronary intervention, aortic valve stenosis, chronic heart failure, and stage 4 chronic kidney disease. He was taking antihypertensive and antiplatelet drugs (100 mg aspirin and 75 mg clopidogrel). Plain computed tomography (CT) of the abdomen and pelvis revealed an incidentally large infrarenal abdominal aortic aneurysm (AAA).

The patient was transferred to our university hospital for further treatment. Contrast-enhanced CT was performed to examine the anatomical features of the AAA. The maximum aneurysm diameter was 55 mm × 65 mm. The proximal neck was 24 mm in diameter and 28 mm in length with an angulation of 40°. The right and left common iliac arteries were 16 mm and 16 mm in diameter, respectively, which were within the instructions for use and anatomically suitable for EVAR. In addition, the left kidney was supplied with double renal arteries, the inferior branch of which originated immediately above the aneurysm. The branch was then sacrificed.

EVAR with the Endurant stent graft (Medtronic Endovascular; Santa Rosa, CA, USA) was performed using a mobile C-arm imaging system. The bilateral common femoral arteries were surgically exposed under sedation and regional anesthesia. A 4F catheter was placed just above the renal arteries through the left brachial artery for intraoperative angiography. The main body (28 mm × 16 mm × 166 mm) was positioned through the left common iliac artery, by which the left inferior renal artery was consequently sacrificed. The main body was extended to the right common iliac artery with a contralateral leg (16 mm × 24 mm × 124 mm). Balloon inflation with a compliant aortic occlusion balloon (Coda Balloon Catheter, Bloomington, IN, USA) was performed in the proximal neck, component junction, and bilateral distal neck but not excessively. Angiography revealed an endoleak in the proximal sealing zone. An aortic cuff was placed inside the proximal neck zone, because the type IV endoleak was indistinguishable from type Ia endoleak. Completion angiography showed that the endoleak had almost disappeared and the procedure was completed.

**Fig. 1:**
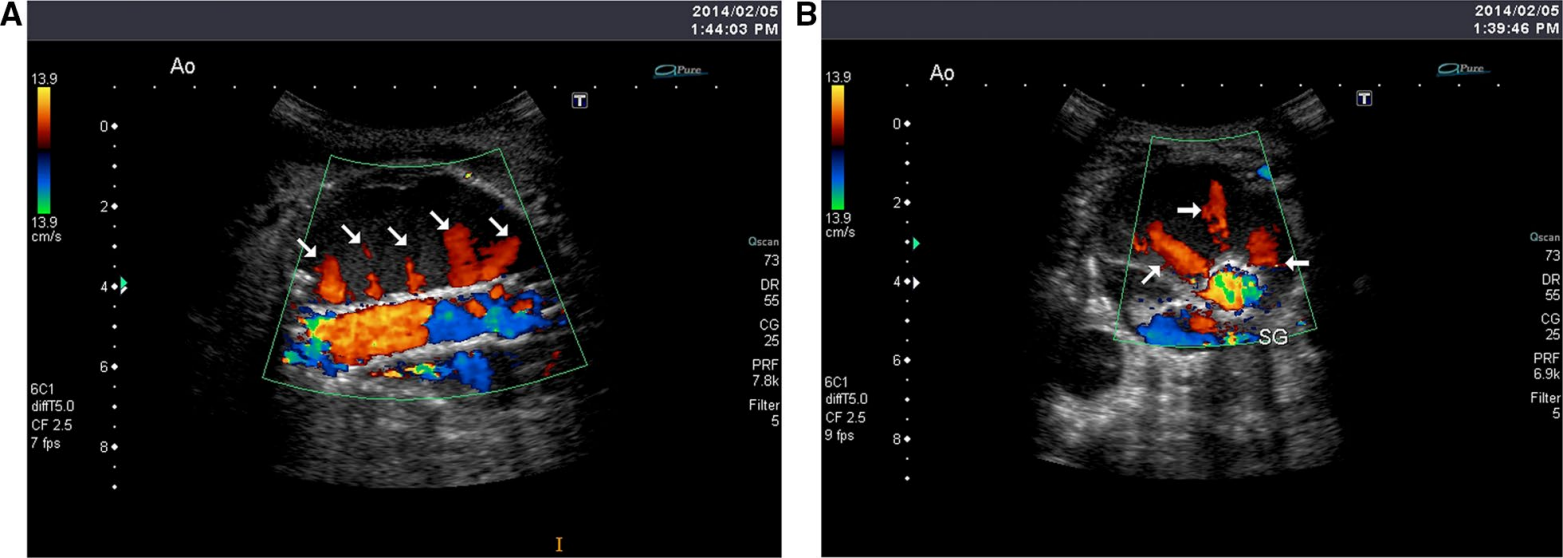
Sagittal (**A**) and axial (**B**) views of the duplex scan showing a regular row of pulsatile blood flow from the main body and left limb

A color duplex ultrasound was performed 7 days after the operation instead of a contrast-enhanced CT because of severe renal insufficiency and revealed a regular row of pulsatile blood flow from the main body and left leg (Additional file 1: Movie 1A and Additional file 2: 1B; Fig. 1A, B). The blood flow appeared to be bleeding from the stent suture lines because of its regularity. Type IV but not type IIIb endoleak was suspected, likely due to stent suture line fabric breakage. However, it was not treated surgically or endovascularly but was followed by CT because of the patient’s poor general health status and the expectation of spontaneous resolution of the leak. The patient was transferred from our hospital to a neighborhood hospital.

**Fig. 2:**
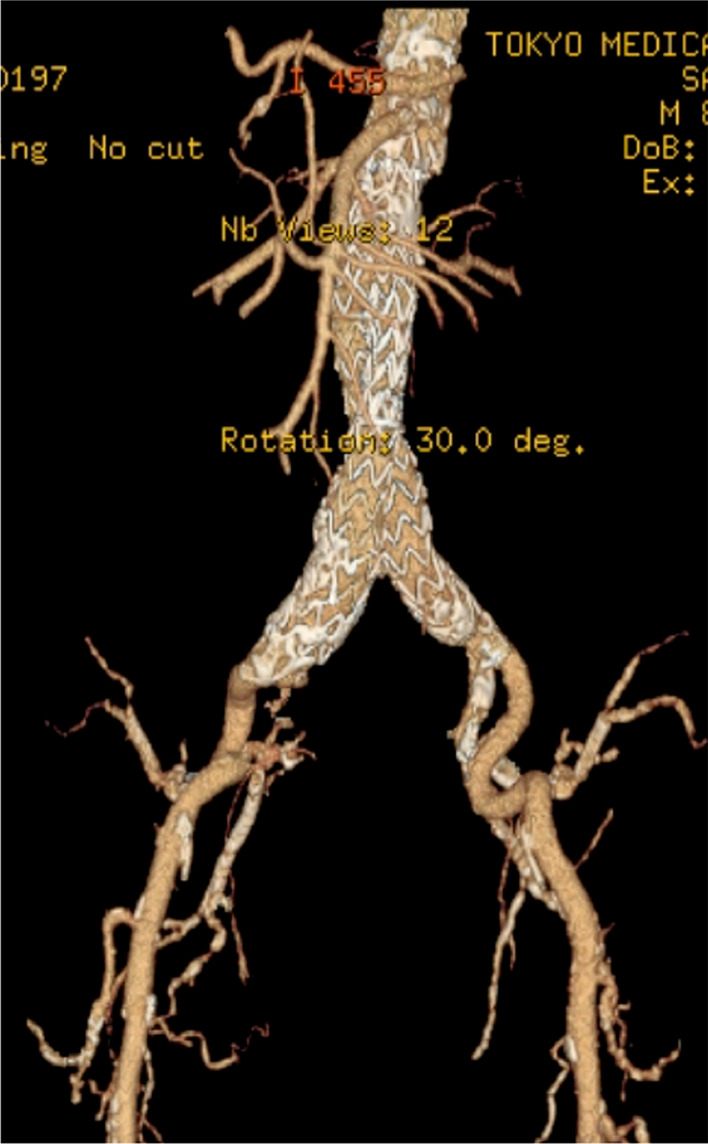
Contrast-enhanced CT showing an oozing of contrast medium on the main body and left limb

Six months later, the patient was readmitted to our hospital because of recurrent fever of unknown origin. Contrast-enhanced CT showed a deformation and enlargement of the aneurysm sac as well as oozing of the contrast medium on the main body and left limb (Fig. 2), suggestive of persistent type IIIb endoleak. However, the patient was considered unsuitable for further surgical intervention because of his deteriorating general health condition. He experienced subdural hematoma due to a fall during his hospital stay and died of uncontrolled subdural hematoma 7 weeks after readmission. Autopsy was performed with the informed consent of his family; it showed no visible abnormal erosion or hole on the graft fabric, suggesting that tiny fabric breakage may have existed along the stent suture line at the time of the manufacturing process.

## Discussion

This case report highlights the occurrence of an early type IIIb endoleak with the Endurant stent graft, which is one of the most commonly used devices in Japan. Although type IIIb endoleaks occur on any devices and at any time after EVAR, they are very rare in the perioperative period [6].

An important issue is the mechanism of fabric defects in early type IIIb endoleaks. The following four hypotheses have been proposed to explain the damage to the fabric: (1) excessive endovascular manipulation; (2) excessive pressure of ballooning; (3) damage to the fabric by the acute tip of a stent displaced by severe neck angulations; and (4) manufacturing defect [6]. In this case, the autopsy showed no visible abnormal erosion or hole on the graft fabric, suggesting that fabric breakage may not have been caused by excessive endovascular manipulation, excessive pressure of ballooning, or acute tip, but may have existed along stent suture lines at the time of the manufacturing process. This is consistent with the report by Matsumura et al. that in a certain graft design, a broken suture line caused a small defect in the graft, leading to “microendoleaks” [7].

Furthermore, we contacted Medtronic, Inc. to ask if this could happen in the Endurant stent graft, but they were not aware of a similar case or provided no explanations.

Type IIIb endoleaks due to suture holes can occur in other commercially available stent grafts, in which metallic stents and polyester grafts are stitched together with sutures. During reoperation due to aneurysmal sac enlargement, intraoperative inspection revealed multiple oozing sites from stent suture line in the Zenith stent graft (Cook Inc, Bloomington, Indiana), the Talent abdominal stent graft (Medtronic, Santa Rosa, CA), and the Valiant thoracic stent graft (Medtronic, Santa Rosa, Calif) [8–10].

Another important issue is the fate of early type IIIb endoleaks. Minor fabric breakage along stent suture lines should not be of any clinical importance; in one report, blood leakage from minor fabric breakage spontaneously resolved in most cases [11]. However, no spontaneous resolution was observed in the report of Matsumura et al., and it did not occur even 6 months after stent graft placement in the present case. Therefore, early type III endoleaks should be treated promptly by endovascular or open surgery, or should be closely monitored if patients might not have been fit for intervention.

In addition, a publication review of type IIIb endoleaks indicated that definitive diagnosis is challenging even with multimodal imaging because of the dynamic nature of detection failure [4, 12]. In this study, color duplex ultrasound clearly demonstrated a regular row of pulsatile blood flow from the main body and left leg, suggesting a type IIIb endoleak likely due to stent suture line breakage. Color duplex ultrasound can provide real-time data in multiple planes, including the longitudinal and circumferential planes, and verify the location and flow characteristics of the endoleak [12]. The use of color duplex ultrasound for surveillance after EVAR will improve the risk management of stent-graft complications.

## Conclusions

This case report describes a rare case of early type IIIb endoleak that is likely due to stent suture line fabric breakage in the Endurant stent graft. Although rare, type IIIb endoleaks can occur even in the perioperative period after EVAR. Early type IIIb endoleaks may not resolve spontaneously and should be treated promptly, if possible.

## Supplementary Information

**Additional file 1: Movie 1A.** Sagittal view of the duplex scan showing a regular row of pulsatile blood flow from the main body and left limb (movie and still images)

**Additional file 2: Movie 1B.** Axial view of the duplex scan showing a regular row of pulsatile blood flow from the main body and left limb (movie and still images).

## Data Availability

Data sharing is not applicable to this article as no data sets were generated or analyzed during the current study.

## Abbreviations

EVAR: Endovascular aneurysm repair
CT: Computed tomography
AAA: Abdominal aortic aneurysm
