# Correction to: Type IIIb endoleak due to stent suture line fabric breakage in the Endurant stent graft: a case report

**DOI:** 10.1186/s40792-022-01443-4

**Published:** 2022-05-11

**Authors:** Satoshi Takahashi, Toshiya Nishibe, Masaki Kano, Shinobu Akiyama, Toru Iwahashi, Hitoshi Ogino

**Affiliations:** Department of Cardiovascular Surgery, Tokyo Medical University, 6-7-1 Nishi-shinjuku, Shinjuku-ku, Tokyo, 160-0023 Japan

**Correction to: Surgical Case Reports (2022) 8:72** https://doi.org/10.1186/s40792-022-01415-8

In the original publication of this article [1], two videos in the additional files are wrong due to a typesetting error, and they were updated.
